# Remote myocardial fibrosis predicts adverse outcome in patients with myocardial infarction on clinical cardiovascular magnetic resonance imaging

**DOI:** 10.1016/j.jocmr.2024.101064

**Published:** 2024-07-23

**Authors:** Nicholas Black, Joshua Bradley, Erik B. Schelbert, Laura J. Bonnett, Gavin A. Lewis, Jakub Lagan, Christopher Orsborne, Pamela F. Brown, Fardad Soltani, Fredrika Fröjdh, Martin Ugander, Timothy C. Wong, Miho Fukui, Joao L. Cavalcante, Josephine H. Naish, Simon G. Williams, Theresa McDonagh, Matthias Schmitt, Christopher A. Miller

**Affiliations:** a Division of Cardiovascular Sciences, School of Medical Sciences, Faculty of Biology, Medicine and Health, Manchester Academic Health Science Centre, University of Manchester, Manchester, UK; b Manchester University NHS Foundation Trust, Manchester, UK; c Department of Medicine, University of Pittsburgh School of Medicine, Pittsburgh, Pennsylvania, USA; d UPMC Cardiovascular Magnetic Resonance Center, Heart and Vascular Institute, Pittsburgh, Pennsylvania, USA; e Clinical and Translational Science Institute, University of Pittsburgh, Pittsburgh, Pennsylvania, USA; f Department of Health Data Science, University of Liverpool, Liverpool, UK; g Royal Liverpool and Broadgreen University Hospitals NHS Trust, Liverpool, UK; h Department of Clinical Physiology, Karolinska University Hospital, and Karolinska Institutet, Stockholm, Sweden; i Minneapolis Heart Institute Foundation, Abbott Northwestern Hospital, Minneapolis, Minnesota, USA; j King’s College Hospital, London, UK; k Wellcome Centre for Cell-Matrix Research, Division of Cell-Matrix Biology & Regenerative Medicine, School of Biology, Faculty of Biology, Medicine & Health, Manchester Academic Health Science Centre, University of Manchester, Manchester, UK; l South Tees NHS Foundation Trust, Middlesbrough, UK; m Kolling Institute, Royal North Shore Hospital, and University of Sydney, Sydney, Australia

**Keywords:** Myocardial infarction, Myocardial fibrosis, Extracellular matrix volume, Deep phenotyping

## Abstract

**Background:**

Heart failure (HF) most commonly occurs in patients who have had a myocardial infarction (MI), but factors other than MI size may be deterministic. Fibrosis of myocardium remote from the MI is associated with adverse remodeling. We aimed to 1) investigate the association between remote myocardial fibrosis, measured using cardiovascular magnetic resonance (CMR) extracellular volume fraction (ECV), and HF and death following MI, 2) identify predictors of remote myocardial fibrosis in patients with evidence of MI and determine the relationship with infarct size.

**Methods:**

Multicenter prospective cohort study of 1199 consecutive patients undergoing CMR with evidence of MI on late gadolinium enhancement. Median follow-up was 1133 (895–1442) days. Cox proportional hazards modeling was used to identify factors predictive of the primary outcome, a composite of first hospitalization for HF (HHF) or all-cause mortality, post-CMR. Linear regression modeling was used to identify determinants of remote ECV.

**Results:**

Remote myocardial fibrosis was a strong predictor of primary outcome (χ^2^: 15.6, hazard ratio [HR]: 1.07 per 1% increase in ECV, 95% confidence interval [CI]: 1.04–1.11, p < 0.001) and was separately predictive of both HHF and death. The strongest predictors of remote ECV were diabetes, sex, natriuretic peptides, and body mass index, but, despite extensive phenotyping, the adjusted model R^2^ was only 0.283. The relationship between infarct size and remote fibrosis was very weak.

**Conclusion:**

Myocardial fibrosis, measured using CMR ECV, is a strong predictor of HHF and death in patients with evidence of MI. The mechanisms underlying remote myocardial fibrosis formation post-MI remain poorly understood, but factors other than infarct size appear to be important.

## 1 Background

Heart failure (HF) is a major and growing public health problem [1]. In the United States and Europe, HF most commonly occurs in patients who have had a myocardial infarction (MI) [2], with up to a third of patients developing HF by 5 years following MI [3].

While adverse left ventricular (LV) remodeling following MI was originally thought to be a mechanical phenomenon resulting from increased wall stress in remote myocardium secondary to the non-contractile infarcted region, it is now clear that other factors are involved. Progressive remodeling occurs in nearly a fifth of patients with small infarcts, yet nearly two-thirds of those with large infarcts do not adversely remodel [4].

Remote myocardial fibrosis is defined by diffuse, disproportionate accumulation of collagen in the interstitium of the non-infarcted myocardium. Numerous preclinical studies have demonstrated remote myocardial fibrosis formation following MI, which is independent of infarct size [5], [6], [7], [8], [9]. Attenuation of remote myocardial fibrosis formation in preclinical work is associated with reduced LV remodeling, improved LV function, reduced incidence of HF, and improved survival [10], [11].

In recent observational clinical studies, acute (2–4 days post-MI) extracellular volume (ECV) expansion in remote myocardium, measured using cardiovascular magnetic resonance (CMR), is independently associated with adverse remodeling, contractile dysfunction, and circulating natriuretic peptide (NP) levels at 3–6 months post-MI [12], [13]. Inhibition of remote myocardial fibrosis formation is widely hypothesized to represent an important mechanism by which angiotensin-converting enzyme inhibitors (ACEi) and mineralocorticoid receptor antagonists reduce the risk of HF and death following MI.

This prospective, multicenter longitudinal cohort study was conducted to 1) investigate whether remote myocardial fibrosis is associated with adverse outcomes, specifically HF and death; and 2) identify predictors of remote myocardial fibrosis in patients with evidence of MI, and in particular, determine the relationship between infarct size and remote fibrosis. Finally, given that LV end-systolic volume index (LVESVi) is considered a key marker of adverse remodeling and outcome post-MI, the dataset was utilized to identify its determinants.

## 2 Methods

### 2.1 Study population

Consecutive patients undergoing CMR at Manchester University NHS Foundation Trust (MFT) between April 1, 2016 and May 31, 2018 and University of Pittsburgh Medical Center (UPMC) between June 1, 2010 and March 25, 2016 were prospectively recruited. Consecutive patients with evidence of MI on late gadolinium enhancement (LGE) were identified. Exclusion criteria included a diagnosis of any of the following: amyloidosis, complex congenital heart disease, Fabry cardiomyopathy, hypertrophic cardiomyopathy, iron overload, myocarditis, and stress-induced cardiomyopathy. Patients were also excluded if their CMR scan was not suitable for analysis. The study was approved by an ethics committee at each site and all participants provided written informed consent (Research Ethics Committee reference 14/NW/1165). The study was prospectively registered on ClinicalTrials.gov (NCT02326324).

### 2.2 Procedures

Data were managed using Research Electronic Data Capture [14]. Baseline characteristic data were determined from patient questionnaires and medical records. CMR was performed using three scanners (1.5T Avanto, 1.5T Espree, and 3T Skyra; Siemens Healthineers; Erlangen, Germany) and included steady-state free precession cine imaging in standard long- and short-axis planes and LGE imaging. T1 mapping (by MOdified Look-Locker Inversion recovery [MOLLI]) was performed before and 15 minutes after gadolinium-based contrast agent administration (gadoterate meglumine [Dotarem; Guerbet, Aulnay-sous-Bois, France] or gadoteridol [ProHance, Bracco Diagnostics, Milan, Italy]) on basal and mid-ventricular short-axis slices. Scan parameters during MOLLI acquisition were typically: voxel size 1.9 × 1.9 × 8 mm^3^, matrix 132 × 192, field-of-view 360 mm, Repetition time/Echo time 293.9/1.01 ms, flip angle 35°, generalized autocalibrating partially parallel acquisition (GRAPPA) acceleration factor 2, GRAPPA reference lines 36, calculated phases 1, segments 66, concatenations 1, bandwidth 1085 Hz/Px, and inversion time 180 ms. CMR image analysis was performed using CVI42 (Circle Cardiovascular Imaging; Calgary, Alberta, Canada). Measurements of ventricular mass, volumetrics, ejection fraction (EF), and atrial area were performed in accordance with Societal Recommendations [15]. Global longitudinal strain (GLS) was measured as described previously for all patients, regardless of the presence of LGE [16]. Ischemic and non-ischemic LGE were quantified visually at each site and were performed in the same way, according to current recommendations [15]. Endocardial and epicardial LV borders were contoured on the short-axis LGE stack and a region of interest was manually contoured around the visually hyper-enhanced myocardium. Infarct size (%) was calculated by dividing the enhanced myocardial mass by the total LV end-diastolic mass and multiplying by 100. Remote myocardial fibrosis was measured using the ECV technique, utilizing basal and mid-LV short-axis T1 maps and same-day hematocrit [17]. Contours utilized the middle third of myocardium to minimize partial voluming of blood. Infarcted myocardium was excluded from the ECV measurement, while foci of non-infarct enhancement were included, as per Societal Recommendations [18]. An illustrative example is shown in Supplementary Fig. 1. Myocardial ECV was calculated as described previously ECV = (1 − hematocrit) [ΔR1myocardium]/[ΔR1bloodpool], where ΔR1 is the difference in relaxation rates (1/T1) pre-contrast and post-contrast [17]. Inter- and intra-observer variability for LGE and ECV quantification were within acceptable limits (Supplementary Appendix). NP levels were laboratory assessed from blood sampling performed on the same day as CMR scanning with N-terminal pro-B-type natriuretic peptide (NT-proBNP) (cobas e 411 immunoanalyzer, Roche Diagnostic, Burgess Hill, UK) or B-type-natriuretic peptide (BNP) (UniCel DxI 800 Access Immunoassay System, Beckman Coulter, Brea, California ). BNP and NT-proBNP track similarly and can be used interchangeably [19]. To facilitate this, NP measurements underwent natural logarithmic transformation before modeling, similar to previous studies [20]. All baseline data collection, including CMR analysis, were performed blinded to outcome status.

### 2.3 Outcomes

The primary outcome was a composite of first hospitalization for HF (HHF) post-CMR or all-cause mortality. Secondary outcomes were 1) first HHF post-CMR and 2) all-cause mortality. Outcome data were collected blinded to patient characteristics.

At MFT, outcome data were obtained from NHS Digital (https://digital.nhs.uk). Hospital Episode Statistics-Admitted Patient Care records were used to identify episodes of hospital admission, and mortality status was derived from Hospital Episode Statistics-Office of National Statistics (Civil registration) data. Similar to previous studies, HHF was defined as the first hospitalization following CMR where HF (ICD-10 I50) was the primary diagnosis [21], [22]. All-cause mortality was classified as all instances of death. The follow-up period at MFT was from the beginning of recruitment (June 1, 2016) until August 19, 2020.

At UPMC, HHF was identified by medical record review using a definition from prior epidemiologic studies [23]. HHF required physician documentation and 1) documented symptoms (e.g. shortness of breath, fatigue, orthopnea, paroxysmal nocturnal dyspnea) and physical signs (e.g. edema, pulmonary rates) consistent with HF; 2) supporting clinical findings (e.g. pulmonary edema on radiography); or 3) therapy for HF, including diuretics, digitalis, ACEi, or beta-blockers [23]. Mortality status was ascertained by Social Security Death Index queries and medical record review. The follow-up period at UPMC was from the beginning of recruitment (June 1, 2010) until April 18, 2017.

### 2.4 Variables

Candidate predictors were selected according to clinical practice and literature review. Twenty-six predictors were selected for prognostic model building, including infarct size and remote fibrosis. During linear model building, variables depicting similar clinical information were not considered.

### 2.5 Statistical analysis

All analyses were performed using R (Version 4.2.0, R Foundation for Statistical Computing, Vienna, Austria).

### 2.6 Missing data

Missing data were rare (1022/49,159; 2.1%) but with a high proportion of incomplete cases (567/1199; 47.2%). Missing data were unintentional, due to incomplete medical records, incomplete CMR scanning, or blood sampling not being performed, thus data were assumed to be missing at random. Multiple imputation by chained equations was used to create 20 imputed datasets [24]. Missing data were imputed from baseline characteristics, outcome status variables, and the Nelson-Aalen estimator of the cumulative hazard for each outcome [25], using predictive mean matching. Results were pooled according to current guidelines [26]. Adjusted R^2^ values were pooled with the Fisher z-transformation [27].

### 2.7 Prognostic modeling

Analysis used Cox proportional hazards modeling on a time since entry timescale to evaluate the relationship between remote fibrosis and the primary and secondary outcomes. For the time to HHF outcome, patients experiencing death were censored at the point of death. For consistency, maximum follow-up in UPMC was truncated at the maximum follow-up in MFT, which was 1565 days.

Continuous variables were assessed for linearity using fractional polynomial transformations in multivariable models, and further investigation with Martingale residuals plots was undertaken for any variables with evidence of non-linearity [28]. The linear form of all continuous variables was preserved. In the presence of multiply imputed data, a composite stepwise selection procedure was utilized to identify the parsimonious multivariable model [24]. First, a stepwise model selection according to Akaike’s Information Criterion was conducted separately in each of the imputed datasets. Variables present in more than 50% of models were then considered for inclusion in the parsimonious model using a D1-statistic test. Wald χ^2^ values represent the strength of association between predictor and outcome and were used to facilitate comparisons between predictors during model building. Investigation of Schoenfeld residuals demonstrated that the models satisfied the proportional hazards assumption [29]. Kaplan-Meier curves are presented.

### 2.8 Linear regression

Linear regression models (univariable and composite stepwise multivariable) were used to assess the relationship between variables and remote myocardial fibrosis, measured using ECV. The relationship between infarct size and remote myocardial fibrosis was plotted. The same methodology was used to identify determinants of LVESVi. Left ventricle ejection fraction (LVEF) was not considered as a candidate predictor of LVESVi, given that LVESVi is used to calculate LVEF.

## 3 Results

### 3.1 Patient characteristics

**Fig. 1:**
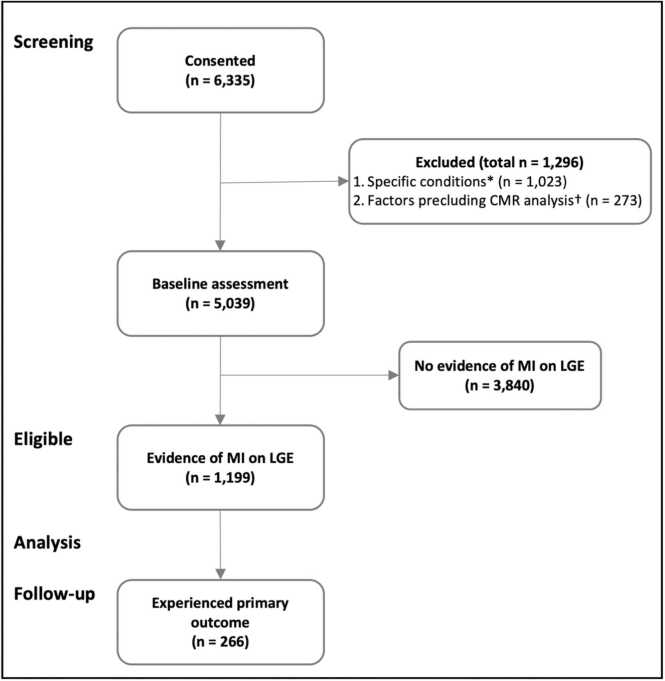
STROBE diagram. *Specific conditions were acute myocarditis (n = 15), amyloidosis (n = 88), complex congenital heart disease (n = 351), Fabry disease (n = 42), hypertrophic cardiomyopathy (n = 487), iron overload (n = 12), and stress-induced cardiomyopathy (n = 28). ^†^Factors precluding cardiovascular magnetic resonance (CMR) analysis were abandoned scanning (n = 59), inadequate image quality (n = 154), and incomplete scan availability (n = 60). STROBE: Strengthening the Reporting of Observational studies in Epidemiology, *MI* myocardial infarction, *LGE* late gadolinium enhancement.

A total of 1199 patients had evidence of MI by LGE imaging (Fig. 1). Baseline characteristics are summarized in Table 1 and Supplemental Table 1. Median age was 64 years (Q1–Q3 55–71 years) and 963 (80%) were male. Six hundred and fifty patients (54.2%) underwent a stress perfusion CMR and nine hundred and fourty seven patients (79.0%) had a scan indication related to coronary artery disease (Supplemental Table 2). Mean infarct size, expressed as a percentage of LV mass, was 13.8 ± 12.5%. Co-existent non-ischemic LGE was present in 141 patients (11.8%). Mean remote myocardial ECV was 27.1 ± 3.8%.

**Table 1:** Baseline characteristics.

	(n = 1199)	Missing (%)
*Demographics*		
Age (years)	63 ± 12	0 (0)
Male (%)	963 [80]	0 (0)
Ethnicity (%)		79 (6.6)
White	1028 (85.7)	
Asian	55 (4.6)	
Black	27 (2.3)	
Other	10 (0.8)	
Body mass index (kg/m^2^)	28.3 [25.2–32.2]	41 (3.4)
Heart failure stage		(0)
Stage A	0 (0)	
Stage B	422 (35.2)	
Stage C	774 (64.6)	
Stage D	3 (0.3)	
Hospitalization status (Inpatient %)	276 (23)	
*Comorbidity*		
Prior hospitalization for heart failure (%)	294 (24.5%)	0 (0)
Previous revascularisation (%)	621 (51.8%)	0 (0)
CABG	238 (19.8)	(0)
PCI	480 (40.0)	(0)
Stroke (%)	85 (7.1%)	0 (0)
Peripheral vascular disease (%)	108 (9.0%)	0 (0)
Diabetes (%)	317 (26.4%)	0 (0)
Hypertension (%)	767 (64.0%)	0 (0)
Raised cholesterol (%)	762 (63.6%)	0 (0)
Chronic obstructive pulmonary disease (%)	113 (9.4%)	0 (0)
History of smoking (%)	727 (60.6%)	0 (0)
Family history of cardiovascular disease (%)	484 (40.4%)	0 (0)
Atrial fibrillation (%)	97 (8.1%)	92 (7.7)
*ECG measurements*		
Heart rate (bpm)	66.5 ± 12.8	99 (8.3)
QRS duration (ms)	102 [93–116]	326 (27.2)
*Laboratory measurements*		
Estimated glomerular filtration rate (mL/min/1.73 m^2^)	79.1 [65.0–90.0]	7 (0.6)
*ln* (natriuretic peptide)	5.8 [4.9–6.8]	250 (20.9)
B-type natriuretic peptide†	217 [97–587]	52/345 (15%) 906/1199 (76%)
N-terminal pro-B-type natriuretic peptide††	420 [162.2–1048.8]	198/854 (23%) 543/1199 (45%)
*CMR characteristics*		
Left ventricle end-diastolic volume index (mL/m^2^)	103 ± 34.8	2 (0.2)
Left ventricle end-systolic volume index (mL/m^2^)	59.1 ± 34.6	2 (0.2)
Left ventricle ejection fraction (%)	46.1 ± 14.5	0 (0)
Left ventricle mass index (g/m^2^)	65.7 ± 20.1	2 (0.2)
Global longitudinal strain (%)	−13.6 ± 4.6	7 (0.6)
Infarct size as percentage of LV mass (%)	10.0 [4.0–20.6]	0 (0)
Non-ischemic LGE as percentage of LV mass, for those with non-ischemic LGE (%)	2.0 [1.0–4.0]	0 (0)
Number of patients with non-ischemic LGE (%)	141 (11.8%)	0 (0)
Remote myocardial extracellular volume fraction (%)	27.1 ± 3.8	100 (8.3)

Values are n (%), mean ± SD, or median (IQR).

*CABG* coronary artery bypass grafting, *CMR* cardiac magnetic resonance, *LGE* late gadolinium enhancement, *LV* left ventricle, *PCI* percutaneous coronary intervention, *ECG* electrocardiogram, *SD* standard deviation, *IQR* interquartile range.

† Patients recruited at the University of Pittsburgh Medical Center (n = 345).

†† Patients recruited at Manchester University NHS Foundation Trust (n = 854).

### 3.2 Predictors of the primary outcome (composite of HHF and death)

Over a median follow-up of 1133 (895–1442) days, the primary outcome occurred in 266 patients, including 126 patients with an HHF, and 183 patients who died.

**Fig. 2:**
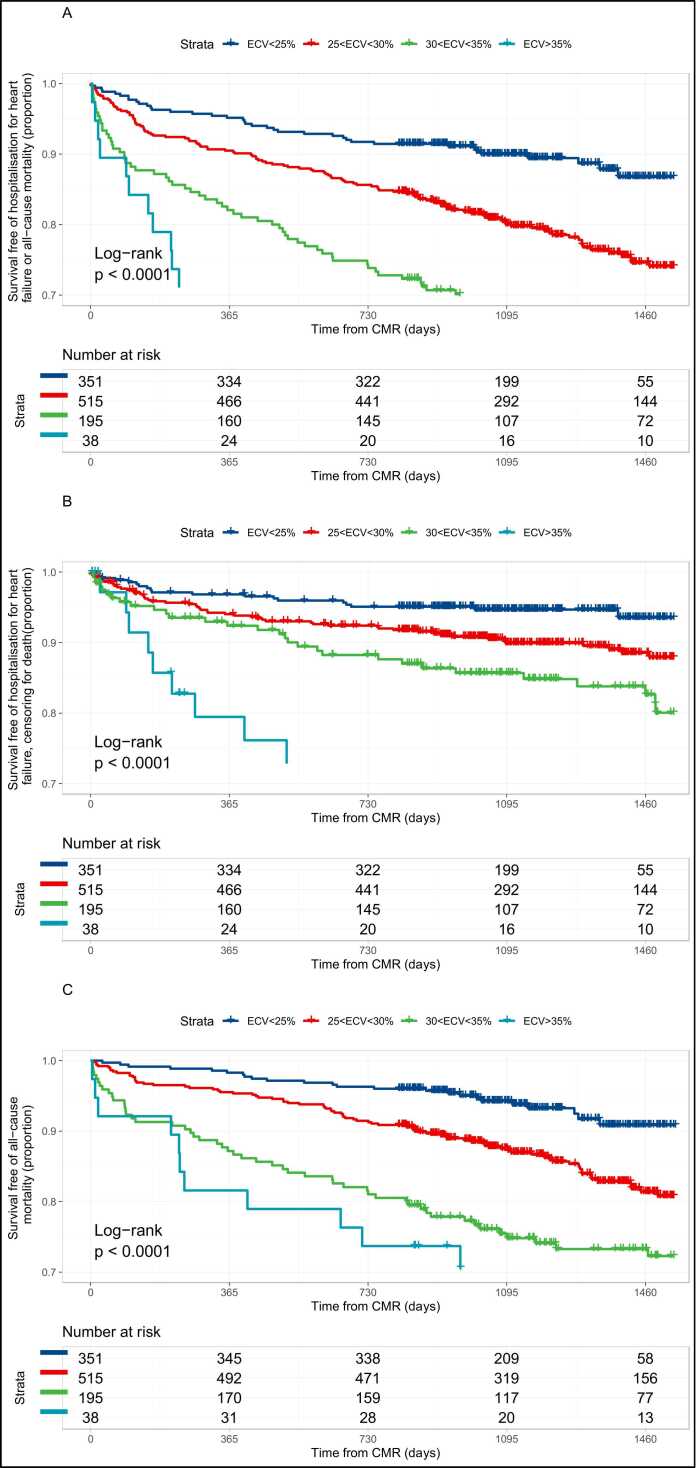
Outcomes according to remote myocardial extracellular volume fraction (ECV) in patients with myocardial infarction. (A) Composite of hospitalization for heart failure and all-cause mortality; (B) hospitalization for heart failure censoring for death; (C) all-cause mortality. n = 1099 patients with complete remote ECV data. *CMR* cardiovascular magnetic resonance.

Remote myocardial fibrosis was a strong predictor of the primary outcome in multivariable analysis (χ^2^: 15.7, hazard ratio [HR]: 1.07 per 1% increase in ECV, 95% confidence interval [CI]: 1.04–1.11, p = 0.00011) (Table 2 and Fig. 2A). Other independent predictors included age, body mass index (BMI), chronic obstructive pulmonary disease, diabetes, family history of cardiovascular disease, history of smoking, *ln* (NP), LVEF, prior HHF and raised cholesterol. LVESVi, infarct size, and non-ischemic LGE were associated with the primary outcome on univariable analysis, but not after multivariable adjustment. Removal of remote myocardial fibrosis from the full model resulted in significantly worse predictive performance (D1-statistic test p < 0.001) and discrimination (Harrell’s C-statistic and 95% CI 0.75 (0.72–0.77) versus 0.77 (0.74–0.79) for the full model, including remote myocardial fibrosis).

**Table 2:** Univariable and multivariable Cox regression models for the outcome of hospitalization for heart failure or all-cause mortality in patients.

Variable	Univariable model					Multivariable model				
	χ^2^	HR	LCL	UCL	p value	χ^2^	HR	LCL	UCL	p value
Age	33.057	1.033	1.022	1.045	<0.001	28.616	1.034	1.021	1.047	<0.001
Atrial fibrillation	4.868	1.538	1.047	2.261	0.0285	.	.	.	.	.
Non-ischemic LGE (%)	19.086	1.072	1.039	1.106	<0.001	.	.	.	.	.
Body mass index	1.126	1.011	0.991	1.031	0.289	16.676	1.043	1.022	1.065	<0.001
COPD	17.017	1.988	1.432	2.761	<0.001	6.393	1.572	1.105	2.236	0.012
Diabetes	38.298	2.162	1.691	2.763	<0.001	21.660	1.864	1.432	2.426	<0.001
eGFR	25.590	0.982	0.975	0.989	<0.001	.	.	.	.	.
Family history of cardiovascular disease	7.077	0.705	0.545	0.913	0.008	3.685	0.771	0.590	1.007	0.056
GLS	119.155	1.165	1.133	1.197	<0.001	.	.	.	.	.
Heart rate	30.268	1.024	1.016	1.033	<0.001	.	.	.	.	.
History of smoking	4.575	1.319	1.022	1.701	0.033	3.718	1.295	0.994	1.687	0.055
Hypertension	0.052	1.030	0.799	1.326	0.820	.	.	.	.	.
Infarct LGE (%)	4.024	1.009	1.000	1.018	0.046	.	.	.	.	.
*ln* (natriuretic peptide)	85.106	1.590	1.439	1.756	<0.001	8.759	1.222	1.068	1.399	0.004
LVEF	105.927	0.957	0.949	0.965	<0.001	31.432	0.972	0.962	0.982	<0.001
LVESVi	78.580	1.011	1.009	1.013	<0.001	.	.	.	.	.
LVMi	39.249	1.016	1.011	1.021	<0.001	.	.	.	.	.
Male sex	0.002	1.007	0.745	1.359	0.966	.	.	.	.	.
Myocardial ECV	87.786	1.135	1.105	1.165	<0.001	15.660	1.074	1.036	1.112	<0.001
Peripheral vascular disease	3.739	1.440	0.993	2.087	0.054	.	.	.	.	.
Prior HHF	38.513	2.195	1.711	2.818	<0.001	4.490	1.337	1.021	1.751	0.035
Prior revascularisation	1.627	0.855	0.672	1.089	0.20	.	.	.	.	.
QRS duration	11.935	1.009	1.004	1.014	<0.001	.	.	.	.	.
Raised cholesterol	4.725	0.763	0.598	0.975	0.031	3.914	0.768	0.591	0.999	0.049
Stroke or TIA	1.438	1.299	0.845	1.997	0.232	.	.	.	.	.
White race	0.119	0.940	0.660	1.339	0.731	.	.	.	.	.

*COPD* chronic obstructive pulmonary disease, *ECV* extracellular volume fraction, *eGFR* estimated glomerular filtration rate, *GLS* global longitudinal strain, *HR* hazard ratio, *HHF* hospitalization for heart failure, *LCL* lower 95% confidence limit, *LVEF* left ventricle ejection fraction, *LVESVi* left ventricle end-systolic volume index, *LVMi* left ventricle mass index, *TIA* transient ischemic attack, *UCL* upper 95% confidence limit, *LGE* late gadolinium enhancement.

Remote myocardial fibrosis remained independently predictive of the primary outcome after the forced inclusion of non-ischemic LGE into the multivariable model (Supplemental Table 3) and the exclusion of inpatients (Supplemental Table 4).

### 3.3 Predictors of secondary outcomes

Remote myocardial fibrosis was also strongly associated with first HHF post-CMR in multivariable modeling (χ^2^: 9.2, HR: 1.07 per 1% increase in ECV, 95% CI: 1.03–1.13, p = 0.0031; Supplemental Table 5 and Fig. 2B), and with death (χ^2^: 11.8, HR: 1.07 per 1% increase in ECV, 95% CI: 1.03–1.12, p = 0.00080; Supplemental Table 6 and Fig. 2C).

### 3.4 Predictors of remote myocardial fibrosis

Univariable associations between baseline characteristics and remote ECV, and the adjusted R^2^ for each variable, are presented in Supplemental Table 7. In multivariable analysis, the strongest predictors of ECV were diabetes, sex, NPs, and BMI (Table 3). The adjusted R^2^ for the multivariable model was 0.283.

**Table 3:** Multivariable linear regression model to predict remote myocardial fibrosis.

	Multivariable model (adjusted R^2^ = 0.283)				
Variable	t	β-Coefficient	LCL	UCL	p value
(Intercept)	29.965	33.605	31.402	35.809	<0.001
Age	−2.014	−0.019	−0.038	0.000	0.045
Body mass index	−4.950	−0.086	−0.121	−0.052	<0.001
COPD	2.022	0.712	0.020	1.403	0.044
Diabetes	7.917	1.834	1.379	2.289	<0.001
Family history of cardiovascular disease	−2.519	−0.536	−0.954	−0.118	0.012
GLS	2.698	0.110	0.030	0.189	0.007
Infarct LGE	−2.841	−0.027	−0.045	−0.008	0.005
*ln* (natriuretic peptide)	5.545	0.529	0.340	0.717	<0.001
LVEF	−3.381	−0.045	−0.071	−0.019	<0.001
Male sex	−7.409	−1.871	−2.366	−1.375	<0.001
Prior revascularisation	−2.401	−0.529	−0.962	−0.096	0.017
Raised cholesterol	−3.132	−0.692	−1.125	−0.258	0.002

*COPD* chronic obstructive pulmonary disease, *GLS* global longitudinal strain, *LCL* lower 95% confidence limit, *LVEF* left ventricle ejection fraction, *UCL* upper 95% confidence limit, *LGE* late gadolinium enhancement.

**Fig. 3:**
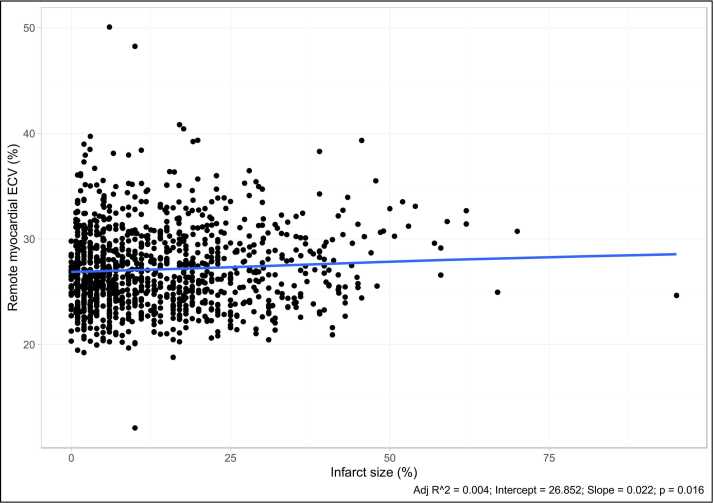
Relationship between myocardial infarction size and remote myocardial extracellular volume (ECV). Points plotted for 1099 participants with complete ECV and late gadolinium enhancement data.

Although there was a statistically significant association between infarct size and remote ECV, the relationship was weak (R^2^ = 0.004, p = 0.016) (Fig. 3).

### 3.5 Predictors of LVESVi

Univariable associations between baseline characteristics and LVESVi, and the adjusted R^2^ for each variable, are presented in Supplemental Table 8. In multivariable analysis, major determinants of LVESVi were GLS, LV mass index, and infarct size (Supplemental Table 8). Multivariable adjusted R^2^ was 0.672 for LVESVi.

## 4 Discussion

The principal findings of this study are that remote myocardial fibrosis, measured using CMR ECV, is a strong predictor for the composite outcome of HHF or death in patients with evidence of MI, and more predictive than infarct size and metrics of left ventricular structure (LVESVi). Moreover, remote myocardial fibrosis is only minimally associated with infarct size, and only 28% of the variance in remote myocardial fibrosis could be explained by deep phenotyping with multivariable regression modeling, suggesting that the mechanisms underlying remote myocardial fibrosis post-MI remain poorly understood.

It has long been recognized that adverse LV remodeling is a major risk factor for deleterious outcomes post-MI, particularly HF and death. Surrogate markers of adverse remodeling, including infarct size, LV dilatation (increase in LVESVi), and LV dysfunction (reduction in LVEF or GLS), are all predictive of worse outcomes post-MI [30], [31], [32], [33]. Moreover, treatments aimed at slowing or potentially preventing these changes, such as renin-angiotensin-aldosterone system (RAAS) inhibitors, have proven efficacious [34], [35]. However, these global measures of LV structure and function do not provide mechanistic insight and occur later in the remodeling process, downstream of the causative pathophysiology. Interestingly in this study, infarct size and LVESVi were not predictive of HHF or death after multivariable adjustment, although a potential explanation is that participants were enrolled without knowing when their index MI occurred. It is therefore possible that participants sustained MI many years previously and variables, such as infarct size and LVESVi become less predictive of adverse outcomes over time. Moreover, patients referred for clinical CMR are subject to referral and survivorship bias, hence infarct size and LVESVi may remain predictive of adverse outcome after multivariable adjustment in all-comers post-MI.

CMR offers unparalleled imaging of myocardial tissue architecture, allowing identification of early substrates of adverse LV remodeling. There is increasing recognition of the role of remote myocardial fibrosis, as measured by ECV expansion in non-infarcted myocardium, in LV remodeling post-MI. Remote ECV expansion occurs within days of acute MI and is associated with adverse remodeling and contractile dysfunction [12], [13]. Moreover, myocardial fibrosis, measured using ECV, is strongly predictive of adverse outcomes in patients with, or at risk of HF without prior HHF, HF with preserved EF, and in unselected patients undergoing CMR [20], [36], [37], [38], [39]. Such cohorts have largely been non-ischemic, with patients with MI comprising only approximately 20% or less. Indeed, the predictive nature of ECV for adverse outcomes in non-ischemic conditions, such as valvular heart disease and cardiomyopathy, is well established [40], [41]. The current study demonstrates that in patients with evidence of MI, remote ECV is highly predictive of the composite outcome of HHF or death. Remote ECV was also one of the few variables to be predictive of HHF and death separately.

Interestingly, univariable regression demonstrated only a very weak association between infarct size and remote ECV (R^2^ = 0.004, p = 0.016). There were unexpected negative associations between remote ECV and several important clinical variables (family history of cardiovascular disease, raised cholesterol, and prior revascularization). We hypothesize that patients with these conditions were more likely to receive optimal medical therapy which in turn may reduce remote ECV. Indeed, the multivariable modeling was only able to explain 28% of the variance in remote ECV (adjusted R^2^ = 0.28), of which diabetes, sex, NPs, and BMI were the most significant variables. Nonetheless, despite extensive phenotyping, most of the variance remains unexplained. We were unable to determine the presence of residual ischemia in patients undergoing a stress perfusion CMR, and it is possible that ischemia in areas remote from infarcted myocardium may contribute toward remote fibrosis. Potentially important variables such as symptom status e.g. New York Heart Association class, medication history, and reason for referral for CMR were not available to include in the multivariable models. In addition, it is hypothesized that there is a genetic predisposition to developing remote myocardial fibrosis [42], [43]. This contrasts with LVESVi, for which regression modeling was able to explain two-thirds of the variance (adjusted R^2^ = 0.67), with infarct, LV mass, and GLS being the most predictive.

If our data are considered in isolation, then remote zone ECV expansion could simply be due to comorbidities. However, preclinical data show that remote zone myocardial fibrosis occurs post-MI and is predictive of subsequent HF and adverse outcomes [44], [45]. Moreover, previous clinical studies have demonstrated that remote zone ECV expansion persists with longer-term follow-up post-MI and predicts adverse LV remodeling [12], [13]. Comorbidities, such as diabetes, appear to affect the amount of remote zone ECV expansion that occurs post-MI [46].

This study raises the possibility that antifibrotic therapies, aimed at inhibiting remote myocardial fibrosis formation, may reduce adverse remodeling and improve outcomes post-MI. For instance, RAAS inhibition, with ACEi and aldosterone antagonists, has been shown to inhibit collagen synthesis, and attenuate and reverse myocardial fibrosis [47], [48], [49]. It remains unclear to what extent the clinical benefits of RAAS inhibition pertain to their impact on myocardial fibrosis versus their blood pressure and afterload effects, although it is clear that their benefit does extend beyond their hemodynamic impact. Pirfenidone, an antifibrotic drug licensed in idiopathic pulmonary fibrosis, significantly reduced myocardial fibrosis in HF with preserved EF [50] but its efficacy in post-MI patients has not been investigated.

The findings of the current study raise the question of what the optimal endpoint of mechanistic trials of antifibrotic interventions post-MI should be. Conventionally, LVESVi has been the most widely applied measurement of remodeling in post-MI phase II trials; however, given its mechanistic insight and much stronger association with outcome, ECV may be more informative.

## 5 Limitations

This study has several limitations. The time interval between prior MI and CMR was not recorded. The mechanism underlying remote ECV expansion in the acute/sub-acute phase following MI (edema/inflammation) is almost certainly different from that underlying remote ECV expansion in the chronic phase (fibrosis), although collagen formation is observed within days of acute MI preclinically [51], [52]. We cannot therefore exclude the possibility that remote ECV may have variable prognostic implications depending on infarct chronicity. Nevertheless, ECV remained strongly and independently predictive of outcomes after excluding patients undergoing CMR as an inpatient (Supplemental Table 4), thus excluding those patients with acute MI. Non-ischemic LGE was included in the remote ECV measurement which would increase the ECV value. CMR scanning was performed on three scanners across two sites, and analyses were performed separately at both sites, which may have resulted in variability in the measurement of remote ECV measurement, although this is reflective of clinical practice. Standardization would be expected to improve the observed predictive value. Similarly, infarct size as a percentage of LV mass was different between the two sites (9.0 vs 14.0% for MFT and UPMC, respectively) which may have been due to inter-observer variability between sites even though the same methodology was used. In addition, endpoints were adjudicated differently between the UK and US cohorts. Previous studies have demonstrated the comprehensiveness of NHS Digital data [21], [53], and it is increasingly being used for trial and registry outcomes [54]. It is of note that the annualized event rates were similar for both sites. Finally, the secondary outcome analyses may be underpowered based on the observed number of events.

## 6 Conclusion

In conclusion, myocardial fibrosis, measured using CMR ECV, was highly predictive for first HHF or death in patients with evidence of MI. The mechanisms underlying remote myocardial fibrosis formation post-MI remain poorly understood, but factors other than infarct size appear to be important.

## Funding

The study was funded by the UK 10.13039/501100000272National Institute for Health Research (NIHR, CS-2015-15-003) and supported by a research grant from Guerbet Laboratories Limited. Immunoassay testing equipment and materials were gifted by Roche Diagnostics International Limited. The work was also supported in part by a British Heart Foundation Accelerator award to The 10.13039/501100000770University of Manchester (BHF, AA/18/4/34221). NIHR, BHF, Guerbet Laboratories Limited, and Roche Diagnostics International Limited had no role in the study design, data collection, data analysis, data interpretation, or writing of the report. The corresponding author had full access to all the data in the study and had final responsibility for the decision to submit for publication. Guerbet Laboratories Limited and Roche Diagnostics International Limited conducted a factual accuracy check of this manuscript, but any decisions to incorporate comments were made solely at the discretion of the authors.

## Author contributions

**Erik B. Schelbert:** Writing – review and editing, Data curation, Conceptualization. **Laura J. Bonnett:** Formal analysis. **Gavin A. Lewis:** Investigation, Formal analysis, Data curation. **Jakub Lagan:** Investigation, Formal analysis, Data curation. **Christopher Orsborne:** Investigation, Formal analysis, Data curation. **Pamela F. Brown:** Investigation, Formal analysis, Data curation. **Fardad Soltani:** Investigation, Formal analysis, Data curation. **Matthias Schmitt:** Supervision. **Christopher A. Miller:** Writing – review and editing, Supervision, Software, Resources, Project administration, Methodology, Investigation, Funding acquisition, Conceptualization. **Fredrika Fröjdh:** Validation, Data curation. **Martin Ugander:** Validation, Data curation. **Timothy C. Wong:** Validation, Data curation. **Nicholas Black:** Writing – review and editing, Writing – original draft, Validation, Investigation. **Miho Fukui:** Validation, Data curation. **Joshua Bradley:** Writing – original draft, Validation, Methodology, Investigation, Formal analysis, Data curation. **Joao L. Cavalcante:** Validation, Data curation. **Josephine H. Naish:** Supervision, Methodology, Investigation. **Simon G. Williams:** Supervision. **Theresa McDonagh:** Supervision.

## Ethics approval and consent

The investigation conforms with the Declaration of Helsinki. The study was approved by an NHS Research Ethics Committee, and all participants provided written informed consent.

## Consent for publication

Not applicable.

## Declaration of competing interests

The authors declare the following financial interests/personal relationships which may be considered as potential competing interests: Christopher Miller reports financial support was provided by National Institute for Health and Care Research. Christopher Miller reports financial support was provided by Guerbet. Christopher Miller reports equipment, drugs, or supplies were provided by Roche. Christopher Miller reports financial support was provided by British Heart Foundation. Christopher Miller reports a relationship with HAYA therapeutics that includes consulting or advisory, PureTech Health that includes consulting or advisory, Amicus Therapeutics Inc that includes funding grants, Roche that includes funding grants, Univar Solutions that includes funding grants, Novartis that includes consulting or advisory, Boehringer Ingelheim Ltd that includes consulting or advisory, Lilly Alliance that includes consulting or advisory, and AstraZeneca PLC that includes consulting or advisory. Erik Schelbert reports a relationship with HAYA therapeutics that includes consulting or advisory, PureTech Health that includes consulting or advisory, and Amicus Therapeutics Inc that includes funding grants and travel reimbursement. Joao L. Cavalcante reports a relationship with Abbott Vascular Inc that includes consulting or advisory and funding grants, Boston Scientific Corp that includes consulting or advisory and funding grants, Edwards Lifesciences Corporation that includes consulting or advisory and funding grants, Medtronic that includes consulting or advisory and funding grants, WL Gore and Associates that includes consulting or advisory and funding grants, Circle Cardiovascular Imaging Inc that includes funding grants, Siemens Healthineers that includes funding grants, 3Mension that includes funding grants, Medis that includes funding grants, and Ziosoft that includes funding grants. Theresa McDonagh reports a relationship with Novartis that includes speaking and lecture fees, AstraZeneca Pharmaceuticals LP that includes speaking and lecture fees, and Vifor Pharma Ltd that includes speaking and lecture fees. Fredrika Fröjdh and Martin Ugander were supported in part by grants from the Swedish Research Council, Swedish Heart and Lung Foundation, Stockholm County Council, and Karolinska Institutet. Martin Ugander is the principal investigator on a research and development agreement regarding cardiovascular magnetic resonance between Siemens and Karolinska University Hospital. Timothy C. Wong was supported by an American Heart Association Scientist Development grant and a Children’s Cardiomyopathy Foundation grant. Josephine Naish has a part-time appointment at Bioxydyn Ltd. The remaining authors have nothing to disclose. The other authors declare that they have no known competing financial interests or personal relationships that could have appeared to influence the work reported in this paper.

## Data Availability

The datasets used and/or analyzed during the current study are available from the corresponding author on reasonable request.
