# Supplementary material for: Remote myocardial fibrosis predicts adverse outcome in patients with myocardial infarction on clinical cardiovascular magnetic resonance imaging
Source: J Cardiovasc Magn Reson. 2024 Jul 23;26(2):101064. doi: 10.1016/j.jocmr.2024.101064 (PMC11347049; doi:10.1016/j.jocmr.2024.101064)
Supplement: Supplementary file 1 — Supplementary material [file mmc1.docx]

**Supplemental Appendix**

[Inter-observer and intra-observer variability in LGE and ECV quantification 3](#_Toc170986590)

[Supplemental Figure 1: Representative contours for ECV measurement. 4](#_Toc170986591)

[4](#_Toc170986592)

[Supplemental Table 1: Table of baseline characteristics by recruitment site 5](#_Toc170986593)

[Supplemental Table 2. CMR scan indications 6](#_Toc170986594)

[Supplemental Table 3. Multivariable Cox regression models for the outcome of hospitalisation for heart failure or all-cause mortality with non-ischaemic LGE forced into the model 7](#_Toc170986595)

[Supplemental Table 4. Multivariable Cox regression for the composite outcome of heart failure hospitalisation and all-cause mortality in outpatients only. 8](#_Toc170986596)

[Supplemental Table 5. Univariable and multivariable Cox regression models for the secondary outcome of hospitalisation for heart failure 9](#_Toc170986597)

[Supplemental Table 6. Univariable and multivariable Cox regression models for the secondary outcome of all-cause mortality 12](#_Toc170986598)

[Supplemental Table 7. Univariable linear regression models to predict remote myocardial fibrosis 14](#_Toc170986599)

[Supplemental Table 8. Univariable and multivariable linear regression models to predict left ventricle end systolic volume index 17](#_Toc170986600)

Inter-observer and intra-observer variability in LGE and ECV quantification

Inter-observer and intra-observer variability were measured at the UK site between authors JB and NB. Scans from twenty patients were used.

For ECV quantification, median and interquartile range (IQR) for inter-observer difference was 0.63 % (0.21 - 1.05) and intra-observer difference was 0.52 % (0.19 - 0.77). Interclass correlation coefficient (ICC) and 95% confidence interval (95% CI) for inter-observer difference was 0.89 (0.74 – 0.96) and for intra-observer difference was 0.97 (0.93 – 0.99).

For LGE quantification, median and IQR for inter-observer difference was 1.29 grams (0.54 – 3.5) and intra-observer difference was 1.46 grams (0.53 – 2.64). ICC and 95% CI for inter-observer difference was 0.96 (0.89-0.98) and intra-observer difference was 0.99 (0.97-1.00).

Supplemental Figure 1: Representative contours for ECV measurement.

Mid slice native T1 map and corresponding LGE image for patient with a) inferolateral infarct and septal non-ischaemic LGE from MFT, b) lateral infarct and septal non-ischaemic LGE from UPMC.

**A)**

**B)**


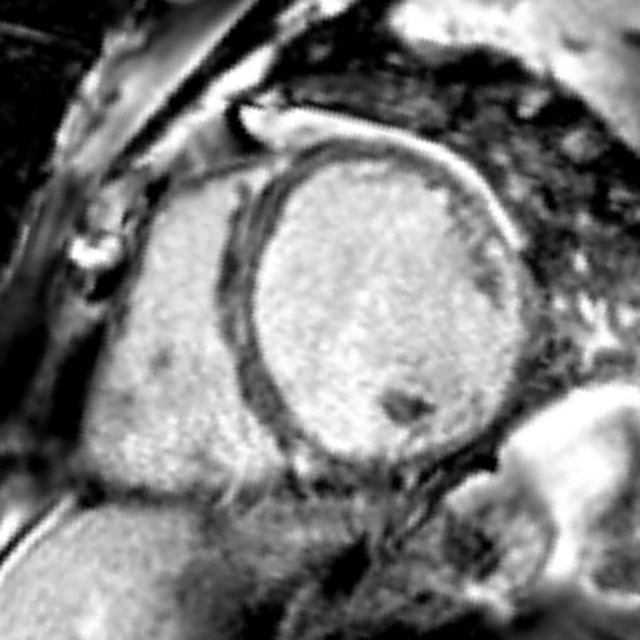

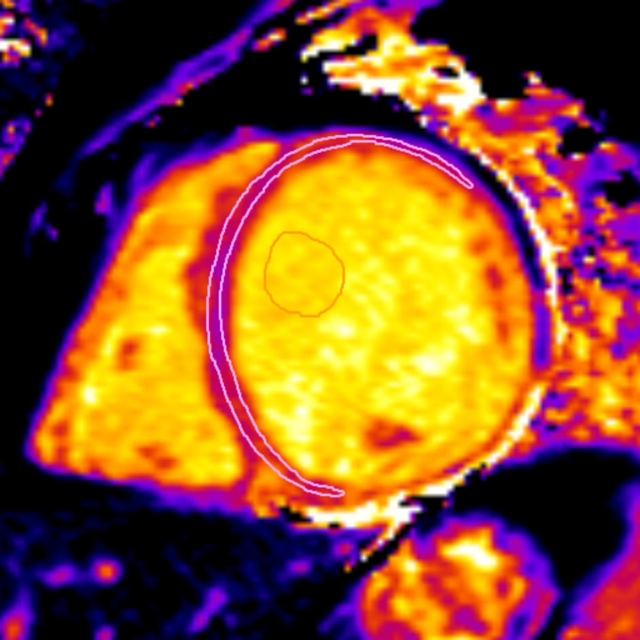

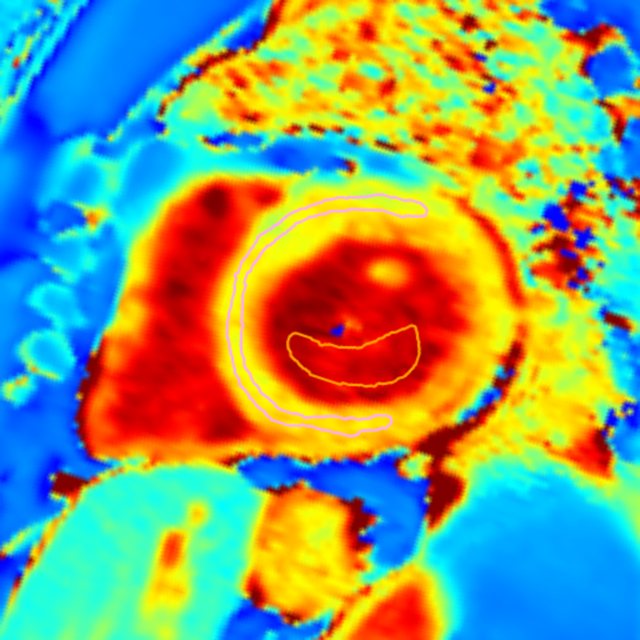

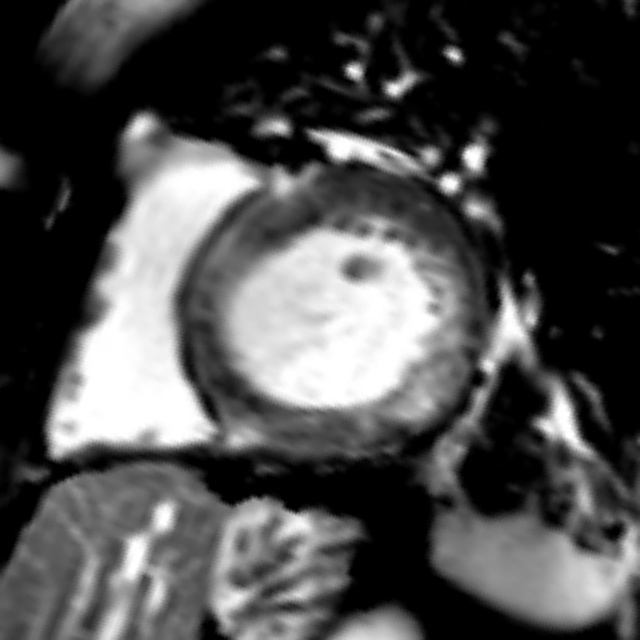


Supplemental Table 1: Table of baseline characteristics by recruitment site

|  | **MFT (n = 854)** | **UPMC (n=345)** |
| --- | --- | --- |
| Age (years) | 63.6 ± 11.3 | 61.6 ± 12.0 |
| Male (%) | 704 (82.4) | 259 (75.1) |
| White ethnicity (%) | 709 (84.2) | 319 (92.5) |
| Body mass index (kg/m^2^) | 28.2 [25.2 – 32.1] | 28.6 [25.3 – 32.6] |
| Previous revascularisation (%) |  |  |
| CABG | 154 (18.0) | 84 (24.3) |
| PCI | 334 (39.1) | 146 (42.3) |
| Stroke (%) | 82 (9.6) | 3 (0.9) |
| Peripheral vascular disease (%) | 75 (8.8) | 33 (9.6) |
| Diabetes (%) | 200 (23.4) | 117 (33.9) |
| Hypertension (%) | 531 (62.2) | 236 (68.4) |
| Raised cholesterol (%) | 567 (66.4) | 195 (56.5) |
| Chronic Obstructive Pulmonary Disease (%) | 82 (9.6) | 31 (9.0) |
| History of smoking (%) | 529 (61.9) | 198 (57.4) |
| Atrial fibrillation (%) | 70 (9.2) | 27 (7.8) |
| Heart rate (bpm) | 64.6 ± 12.0 | 73.1 ± 13.6 |
| QRS duration (ms) | 102 [93 – 116] | 98 [88 – 114] |
| Estimated glomerular filtration rate (mL/min/1.73m^2^) | 80.0 [65.0 – 90.0] | 79 [63 – 91.2] |
| *ln* (natriuretic peptide) | 6.0 [5.1 – 7.0] | 5.4 [4.6 – 6.4] |
| Left ventricle end diastolic volume index (mL/m^2^) | 102.9 ± 33.6 | 103.4 ± 37.7 |
| Left ventricle end systolic volume index (mL/m^2^) | 57.2 ± 32.9 | 63.8 ± 38.2 |
| Left ventricle ejection fraction (%) | 47.7 ± 13.8 | 42.2 ± 15.4 |
| Left ventricle mass index (g/m^2^) | 64.7 ± 19.2 | 68.3 ± 22.2 |
| Global longitudinal strain (%) | -14.4 ± 4.5 | -11.7 ± 4.4 |
| Infarct size as percentage of LV mass (%) | 9.0 [4.0 – 18.0] | 14.0 [5.1 – 25.7] |
| Non-ischaemic LGE as percentage of LV mass, for those with non-ischaemic LGE (%) | 2.0 [1.0 – 4.0] | 2.2 [0.8 – 6.0] |
| Number of patients with non-ischaemic LGE (%) | 108 (12.6 %) | 33 (9.6 %) |
| Remote myocardial extracellular volume fraction (%) | 26.4 ± 3.7 | 28.7 ± 3.6 |

Values are n (%), mean ± SD, or median (IQR).

CABG=coronary artery bypass grafting, CMR=cardiac magnetic resonance, LGE=late gadolinium enhancement, LV=left ventricle, PCI=percutaneous coronary intervention.

Supplemental Table 2. CMR scan indications

| **Scan indications** | **Number of patients (%)** |
| --- | --- |
| Ischaemic heart disease | 947 (79.0) |
| Arrhythmia | 113 (9.4) |
| Cardiac mass | 66 (5.5) |
| Heart failure | 57 (4.8) |
| Pericardial disease | 45 (3.8) |
| Cardiomyopathy | 35 (2.9) |
| Valvular heart disease | 33 (2.8) |
| Aortic disease | 13 (1.1) |

Patients listed for more than one scan indication hence total number of scan indications (1,309) exceeds total number of patients (1,199).

Supplemental Table 3. Multivariable Cox regression models for the outcome of hospitalisation for heart failure or all-cause mortality with non-ischaemic LGE forced into the model

| **Variable** | **All patients (N=1199)** | | | | |
| --- | --- | --- | --- | --- | --- |
|  | **χ^2^** | **HR** | **LCL** | **UCL** | **p value** |
| Age | 27.471 | 1.033 | 1.021 | 1.046 | <0.001 |
| BMI | 16.823 | 1.043 | 1.022 | 1.065 | <0.001 |
| Raised cholesterol | 4.044 | 0.765 | 0.588 | 0.995 | 0.045 |
| COPD | 6.608 | 1.585 | 1.114 | 2.255 | 0.011 |
| Diabetes | 22.270 | 1.880 | 1.444 | 2.446 | <0.001 |
| Family history of cardiovascular disease | 3.946 | 0.763 | 0.584 | 0.998 | 0.048 |
| History of smoking | 4.022 | 1.310 | 1.005 | 1.709 | 0.046 |
| LVEF | 30.840 | 0.972 | 0.962 | 0.982 | <0.001 |
| *ln* (natriuretic peptide) | 8.819 | 1.222 | 1.069 | 1.399 | 0.004 |
| Myocardial ECV | 14.093 | 1.070 | 1.033 | 1.109 | <0.001 |
| Non-ischaemic LGE | 1.157 | 1.021 | 0.983 | 1.061 | 0.283 |
| Prior HHF | 4.220 | 1.326 | 1.012 | 1.739 | 0.041 |

Abbreviations as per previous tables

Supplemental Table 4. Multivariable Cox regression for the composite outcome of heart failure hospitalisation and all-cause mortality in outpatients only.

| **Variable** | **Outpatients (N=918)** | | | | |
| --- | --- | --- | --- | --- | --- |
|  | **χ^2^** | **HR** | **LCL** | **UCL** | **p value** |
| Age | 24.392 | 1.042 | 1.026 | 1.058 | <0.001 |
| Atrial fibrillation | 3.374 | 0.611 | 0.086 | 1.137 | 0.069 |
| BMI | 9.928 | 1.041 | 1.016 | 1.066 | 0.002 |
| Raised cholesterol | 5.304 | 0.679 | 0.349 | 1.008 | 0.023 |
| COPD | 4.242 | 1.575 | 1.143 | 2.006 | 0.041 |
| Diabetes | 4.585 | 1.454 | 1.111 | 1.797 | 0.034 |
| Family history of cardiovascular disease | 4.61 | 0.700 | 0.375 | 1.025 | 0.033 |
| Heart rate | 5.655 | 1.015 | 1.003 | 1.028 | 0.019 |
| History of smoking | 3.041 | 1.344 | 1.012 | 1.676 | 0.083 |
| LVEF | 19.433 | 0.972 | 0.959 | 0.984 | <0.001 |
| *ln* (natriuretic peptide) | 10.223 | 1.265 | 1.121 | 1.409 | 0.002 |
| Myocardial ECV | 12.323 | 1.077 | 1.036 | 1.119 | <0.001 |
| Prior HHF | 5.635 | 1.491 | 1.161 | 1.821 | 0.019 |

Abbreviations as per previous tables

Supplemental Table 5. Univariable and multivariable Cox regression models for the secondary outcome of hospitalisation for heart failure

| **Variable** | **Univariable model** | | | | |  | **Multivariable model** | | | | |
| --- | --- | --- | --- | --- | --- | --- | --- | --- | --- | --- | --- |
|  | **χ^2^** | **HR** | **LCL** | **UCL** | **p value** |  | **χ^2^** | **HR** | **LCL** | **UCL** | **p value** |
| Age | 7.925 | 1.023 | 1.007 | 1.040 | 0.006 |  | 6.940 | 1.024 | 1.006 | 1.042 | 0.010 |
| Atrial fibrillation | 8.174 | 2.036 | 1.244 | 3.334 | 0.005 |  | ·· | ·· | ·· | ·· | ·· |
| Non-ischaemic LGE | 7.118 | 1.066 | 1.017 | 1.118 | 0.009 |  | ·· | ·· | ·· | ·· | ·· |
| Body mass index | 2.230 | 1.021 | 0.993 | 1.050 | 0.138 |  | 12.988 | 1.056 | 1.025 | 1.088 | <0.001 |
| COPD | 0.340 | 1.186 | 0.664 | 2.119 | 0.561 |  | ·· | ·· | ·· | ·· | ·· |
| Diabetes | 17.415 | 2.129 | 1.488 | 3.046 | <0.001 |  | 4.955 | 1.535 | 1.048 | 2.247 | 0.028 |
| eGFR | 13.420 | 0.981 | 0.971 | 0.991 | <0.001 |  | ·· | ·· | ·· | ·· | ·· |
| Family history of cardiovascular disease | 1.501 | 0.795 | 0.549 | 1.152 | 0.223 |  | ·· | ·· | ·· | ·· | ·· |
| GLS | 86.444 | 1.215 | 1.166 | 1.267 | <0.001 |  | ·· | ·· | ·· | ·· | ·· |
| Heart rate | 13.883 | 1.024 | 1.011 | 1.038 | <0.001 |  | ·· | ·· | ·· | ·· | ·· |
| History of smoking | 1.684 | 1.275 | 0.880 | 1.845 | 0.197 |  | ·· | ·· | ·· | ·· | ·· |
| hypertension | 0.040 | 0.964 | 0.668 | 1.390 | 0.843 |  | ·· | ·· | ·· | ·· | ·· |
| Infarct LGE | 5.399 | 1.015 | 1.002 | 1.027 | 0.022 |  | ·· | ·· | ·· | ·· | ·· |
| *ln* (natriuretic peptide) | 47.308 | 1.662 | 1.434 | 1.925 | <0.001 |  | 4.025 | 1.214 | 1.001 | 1.473 | 0.049 |
| LVEF | 91.086 | 0.940 | 0.928 | 0.952 | <0.001 |  | 38.602 | 0.952 | 0.937 | 0.967 | <0.001 |
| LVESVi | 79.612 | 1.014 | 1.011 | 1.017 | <0.001 |  | ·· | ·· | ·· | ·· | ·· |
| LVMi | 21.321 | 1.016 | 1.009 | 1.024 | <0.001 |  | ·· | ·· | ·· | ·· | ·· |
| Male sex | 0.016 | 1.029 | 0.661 | 1.601 | 0.899 |  | ·· | ·· | ·· | ·· | ·· |
| Myocardial ECV | 50.383 | 1.140 | 1.099 | 1.183 | <0.001 |  | 9.217 | 1.074 | 1.025 | 1.126 | 0.003 |
| Peripheral vascular disease | 0.000 | 0.999 | 0.535 | 1.867 | 0.998 |  | ·· | ·· | ·· | ·· | ·· |
| Prior HHF | 53.013 | 3.679 | 2.582 | 5.242 | <0.00001 |  | 15.433 | 2.117 | 1.450 | 3.090 | <0.001 |
| Prior revascularisation | 0.126 | 0.939 | 0.660 | 1.336 | 0.723 |  | 2.948 | 1.405 | 0.949 | 2.079 | 0.089 |
| QRS | 11.965 | 1.013 | 1.005 | 1.020 | 0.001 |  | ·· | ·· | ·· | ·· | ·· |
| Raised cholesterol | 2.198 | 0.765 | 0.535 | 1.094 | 0.141 |  | ·· | ·· | ·· | ·· | ·· |
| Stroke or TIA | 0.034 | 0.935 | 0.453 | 1.928 | 0.854 |  | ·· | ·· | ·· | ·· | ·· |
| White race | 1.604 | 0.738 | 0.459 | 1.186 | 0.208 |  | 4.434 | 0.596 | 0.366 | 0.970 | 0.037 |

COPD=chronic obstructive pulmonary disease, ECV=extracellular volume, eGFR=estimated glomerular filtration rate, GLS=global longitudinal strain, HHF=hospitalisation for heart failure, HR=hazard ratio, LVEF=left ventricle ejection fraction, LCL=lower confidence limit, LVMi=left ventricle mass index, LVESVi=left ventricle end systolic volume index, PVD=peripheral vascular disease, TIA=transient ischaemic attack, UCL=upper confidence limit

Supplemental Table 6. Univariable and multivariable Cox regression models for the secondary outcome of all-cause mortality

| **Variable** | **Univariable model** | | | | |  | **Multivariable model** | | | | |
| --- | --- | --- | --- | --- | --- | --- | --- | --- | --- | --- | --- |
|  | **χ^2^** | **HR** | **LCL** | **UCL** | **p value** |  | **χ^2^** | **HR** | **LCL** | **UCL** | **p value** |
| Age | 40.287 | 1.046 | 1.031 | 1.061 | <0.001 |  | 27.159 | 1.040 | 1.025 | 1.056 | <0.001 |
| Atrial fibrillation | 1.728 | 1.369 | 0.854 | 2.196 | 0.191 |  | **··** | **··** | **··** | **··** | **··** |
| Non-ischaemic LGE | 22.801 | 1.086 | 1.050 | 1.124 | <0.001 |  | 3.832 | 1.042 | 1.000 | 1.086 | 0.052 |
| BMI | 0.034 | 1.002 | 0.977 | 1.028 | 0.855 |  | 5.993 | 1.032 | 1.006 | 1.059 | 0.015 |
| COPD | 18.427 | 2.283 | 1.562 | 3.337 | <0.001 |  | 9.590 | 1.841 | 1.248 | 2.717 | 0.002 |
| Diabetes | 27.118 | 2.179 | 1.622 | 2.926 | <0.001 |  | 14.127 | 1.792 | 1.319 | 2.434 | <0.001 |
| eGFR | 18.701 | 0.982 | 0.973 | 0.990 | <0.001 |  | **··** | **··** | **··** | **··** | **··** |
| Family history of cardiovascular disease | 6.631 | 0.661 | 0.482 | 0.908 | 0.011 |  | 4.883 | 0.693 | 0.499 | 0.962 | 0.028 |
| GLS | 59.617 | 1.138 | 1.101 | 1.177 | <0.001 |  | 11.561 | 1.070 | 1.029 | 1.113 | 0.001 |
| Heart rate | 22.016 | 1.025 | 1.015 | 1.036 | <0.001 |  | **··** | **··** | **··** | **··** | **··** |
| History of smoking | 2.556 | 1.282 | 0.943 | 1.743 | 0.112 |  | **··** | **··** | **··** | **··** | **··** |
| Hypertension | 0.695 | 1.140 | 0.836 | 1.556 | 0.406 |  | **··** | **··** | **··** | **··** | **··** |
| Infarct LGE | 0.505 | 1.004 | 0.993 | 1.015 | 0.478 |  | **··** | **··** | **··** | **··** | **··** |
| *ln* (natriuretic peptide) | 55.454 | 1.544 | 1.375 | 1.732 | <0.001 |  | 6.604 | 1.216 | 1.045 | 1.415 | 0.012 |
| LVEF | 39.536 | 0.968 | 0.959 | 0.978 | <0.001 |  | **··** | **··** | **··** | **··** | **··** |
| LVESVi | 19.721 | 1.008 | 1.004 | 1.011 | <0.001 |  | **··** | **··** | **··** | **··** | **··** |
| LVMi | 19.307 | 1.014 | 1.007 | 1.020 | <0.001 |  | **··** | **··** | **··** | **··** | **··** |
| Male sex | 0.011 | 0.981 | 0.685 | 1.406 | 0.917 |  | **··** | **··** | **··** | **··** | **··** |
| Myocardial ECV | 57.155 | 1.131 | 1.095 | 1.169 | <0.001 |  | 11.782 | 1.074 | 1.031 | 1.119 | 0.001 |
| Peripheral vascular disease | 9.053 | 1.872 | 1.241 | 2.825 | 0.003 |  | **··** | **··** | **··** | **··** | **··** |
| Prior HHF | 5.533 | 1.459 | 1.063 | 2.003 | 0.020 |  | **··** | **··** | **··** | **··** | **··** |
| Prior revascularisation | 0.721 | 0.882 | 0.659 | 1.181 | 0.397 |  | **··** | **··** | **··** | **··** | **··** |
| QRS duration | 3.147 | 1.006 | 0.999 | 1.013 | 0.079 |  | **··** | **··** | **··** | **··** | **··** |
| Raised cholesterol | 2.811 | 0.778 | 0.579 | 1.045 | 0.095 |  | **··** | **··** | **··** | **··** | **··** |
| Stroke or TIA | 3.924 | 1.617 | 1.002 | 2.609 | 0.049 |  | **··** | **··** | **··** | **··** | **··** |
| White race | 0.143 | 1.090 | 0.695 | 1.708 | 0.706 |  | **··** | **··** | **··** | **··** | **··** |

Abbreviations as per previous table.

Supplemental Table 7. Univariable linear regression models to predict remote myocardial fibrosis

|  | **Univariable model** | | | | | | |
| --- | --- | --- | --- | --- | --- | --- | --- |
| **Variable** | **t** | **Intercept** | **β-Coefficient** | **LCL** | **UCL** | **p value** | **Adjusted R^2^** |
| (Intercept) | ·· | ·· | ·· | ·· | ·· | ·· | ·· |
| Age | 0.670 | 26.727 | 0.007 | -0.013 | 0.027 | 0.503 | NA |
| Atrial fibrillation | 3.214 | 27.032 | 1.339 | 0.520 | 2.158 | 0.001 | 0.009 |
| Non-ischaemic LGE | 4.533 | 27.030 | 0.315 | 0.177 | 0.453 | <0.001 | 0.027 |
| Body mass index | -4.380 | 29.613 | -0.084 | -0.122 | -0.046 | <0.001 | 0.016 |
| COPD | 2.799 | 27.052 | 1.101 | 0.329 | 1.874 | 0.005 | 0.006 |
| Diabetes | 6.050 | 26.749 | 1.538 | 1.039 | 2.037 | <0.001 | 0.030 |
| eGFR | -1.453 | 27.889 | -0.010 | -0.022 | 0.003 | 0.147 | 0.001 |
| Family history of cardiovascular disease | -4.081 | 27.547 | -0.969 | -1.436 | -0.503 | <0.001 | 0.014 |
| GLS | 12.354 | 31.179 | 0.295 | 0.248 | 0.342 | <0.001 | 0.124 |
| Heart rate | 8.469 | 22.040 | 0.076 | 0.059 | 0.094 | <0.001 | 0.066 |
| History of smoking | 0.172 | 27.131 | 0.040 | -0.421 | 0.502 | 0.864 | NA |
| Hypertension | -1.232 | 27.345 | -0.296 | -0.767 | 0.175 | 0.218 | NA |
| Infarct LGE | 2.405 | 26.852 | 0.022 | 0.004 | 0.040 | 0.016 | 0.004 |
| *ln* (natriuretic peptide) | 10.241 | 21.793 | 0.914 | 0.738 | 1.090 | <0.001 | 0.111 |
| LVEF | -12.127 | 31.360 | -0.091 | -0.106 | -0.076 | <0.001 | 0.117 |
| LVESVi | 11.161 | 25.038 | 0.036 | 0.030 | 0.042 | <0.001 | 0.103 |
| LVMi | 6.298 | 24.816 | 0.036 | 0.025 | 0.047 | <0.001 | 0.034 |
| Male sex | -6.183 | 28.553 | -1.740 | -2.292 | -1.188 | <0.001 | 0.032 |
| Peripheral vascular disease | 1.303 | 27.107 | 0.538 | -0.273 | 1.348 | 0.193 | NA |
| Prior HHF | 2.581 | 26.983 | 0.703 | 0.168 | 1.237 | 0.010 | 0.005 |
| Prior revascularisation | -6.005 | 27.867 | -1.373 | -1.821 | -0.924 | <0.001 | 0.031 |
| QRS duration | 4.182 | 24.582 | 0.024 | 0.013 | 0.035 | <0.001 | 0.020 |
| Raised cholesterol | -4.999 | 27.910 | -1.187 | -1.654 | -0.721 | <0.001 | 0.021 |
| Stroke or TIA | 0.700 | 27.131 | 0.341 | -0.619 | 1.301 | 0.485 | NA |
| White race | 0.826 | 26.914 | 0.280 | -0.385 | 0.945 | 0.409 | NA |

NA=not applicable, LCL=lower confidence limit, UCL=upper confidence limit. Other abbreviations as per previous tables.

Supplemental Table 8. Univariable and multivariable linear regression models to predict left ventricle end systolic volume index

|  | **Univariable model** | | | | | | |  | **Multivariable model (Adjusted R^2^ = 0.672)** | | | | |
| --- | --- | --- | --- | --- | --- | --- | --- | --- | --- | --- | --- | --- | --- |
| **Variable** | **t** | **Intercept** | **β-Coefficient** | **LCL** | **UCL** | **p value** | **Adjusted R^2^** |  | **t** | **β-Coefficient** | **LCL** | **UCL** | **p value** |
| (Intercept) | **··** | **··** | **··** | **··** | **··** | **··** | **··** |  | 5.257 | 60.936 | 38.162 | 83.709 | <0.001 |
| Age | -0.819 | 63.601 | -0.071 | -0.241 | 0.099 | 0.413 | NA |  | -5.291 | -0.321 | -0.440 | -0.202 | <0.001 |
| Atrial fibrillation | 1.124 | 58.738 | 4.240 | -3.172 | 11.652 | 0.261 | NA |  | **··** | **··** | **··** | **··** | **··** |
| Non-ischaemic LGE | 3.294 | 58.490 | 1.607 | 0.650 | 2.564 | 0.001 | 0.008 |  | **··** | **··** | **··** | **··** | **··** |
| Body mass index | -4.005 | 79.018 | -0.680 | -1.013 | -0.347 | <0.001 | 0.013 |  | -2.961 | -0.314 | -0.522 | -0.106 | 0.003 |
| COPD | -0.669 | 59.345 | -2.290 | -9.008 | 4.428 | 0.504 | NA |  | -4.038 | -8.301 | -12.336 | -4.267 | <0.001 |
| Diabetes | -0.020 | 59.141 | -0.046 | -4.498 | 4.406 | 0.984 | NA |  | ·· | ·· | ·· | ·· | ·· |
| eGFR | -0.562 | 61.599 | -0.032 | -0.145 | 0.080 | 0.574 | NA |  | 1.703 | 0.062 | -0.009 | 0.133 | 0.089 |
| Family history of cardiovasc-ular disease | -2.661 | 61.314 | -5.412 | -9.403 | -1.422 | 0.008 | 0.005 |  | 2.372 | 2.903 | 0.502 | 5.304 | 0.018 |
| GLS | 35.829 | 132.888 | 5.413 | 5.117 | 5.710 | <0.001 | 0.517 |  | 16.689 | 3.285 | 2.899 | 3.672 | <0.001 |
| Heart rate | 3.975 | 37.690 | 0.320 | 0.162 | 0.478 | <0.001 | 0.014 |  | -4.050 | -0.216 | -0.321 | -0.111 | <0.001 |
| History of smoking | 1.867 | 56.812 | 3.821 | -0.194 | 7.837 | 0.062 | 0.013 |  | **··** | **··** | **··** | **··** | **··** |
| Hypertension | -4.104 | 64.568 | -8.502 | -12.566 | -4.438 | <0.001 | 0.130 |  | -2.969 | -3.764 | -6.252 | -1.277 | 0.003 |
| Infarct LGE | 13.416 | 45.284 | 1.001 | 0.855 | 1.147 | <0.001 | 0.173 |  | 8.578 | 0.466 | 0.359 | 0.572 | 0.001 |
| *ln* (natriuretic peptide) | 14.763 | -1.115 | 10.263 | 8.898 | 11.629 | <0.001 | 0.313 |  | 3.535 | 2.228 | 0.982 | 3.474 | <0.001 |
| LVMi | 23.375 | -4.123 | 0.962 | 0.882 | 1.043 | <0.001 | 0.009 |  | 12.250 | 0.452 | 0.379 | 0.524 | <0.001 |
| Male sex | 3.361 | 52.365 | 8.421 | 3.506 | 13.336 | 0.001 | 0.103 |  | **··** | **··** | **··** | **··** | **··** |
| Myocardial ECV | 11.046 | -19.720 | 2.904 | 2.387 | 3.420 | <0.001 | NA |  | 2.912 | 0.544 | 0.177 | 0.911 | 0.004 |
| Peripheral vascular disease | -0.365 | 59.244 | -1.276 | -8.133 | 5.581 | 0.715 | 0.051 |  | **··** | **··** | **··** | **··** | **··** |
| Prior HHF | 8.090 | 54.635 | 18.325 | 13.881 | 22.769 | <0.001 | 0.040 |  | 4.397 | 6.365 | 3.524 | 9.206 | <0.001 |
| Prior revascularisation | -7.140 | 66.384 | -14.009 | -17.858 | -10.159 | <0.001 | 0.162 |  | -5.271 | -6.541 | -8.977 | -4.106 | <0.001 |
| QRS duration | 11.585 | -5.107 | 0.593 | 0.491 | 0.695 | <0.001 | 0.013 |  | 5.434 | 0.203 | 0.129 | 0.278 | <0.001 |
| Raised cholesterol | -4.163 | 64.595 | -8.600 | -12.654 | -4.547 | <0.001 | 0.002 |  | **··** | **··** | **··** | **··** | **··** |
| Stroke or TIA | -0.305 | 59.214 | -1.197 | -8.890 | 6.495 | 0.760 | NA |  | **··** | **··** | **··** | **··** | **··** |
| White race | -0.173 | 59.569 | -0.509 | -6.285 | 5.268 | 0.863 | NA |  | **··** | **··** | **··** | **··** | **··** |

Abbreviations as per previous tables.
